# Supplementary material for: NGF-Enhanced Vasculogenic Properties of Epithelial Ovarian Cancer Cells Is Reduced by Inhibition of the COX-2/PGE2 Signaling Axis
Source: Cancers (Basel). 2019 Dec 7;11(12):1970. doi: 10.3390/cancers11121970 (PMC6966471; doi:10.3390/cancers11121970)
Supplement: Supplementary file 1 [file cancers-11-01970-s001.zip › cancers-640510-Supplementary material.pdf]

Supplementary Materials: NGF-Enhanced Vasculogenic Properties of Epithelial Ovarian Cancer cells is Reduced by Inhibition of the COX-2/PGE$_2$ Signaling Axis

Maritza P. Garrido, Iván Hurtado, Manuel Valenzuela, Renato Salvatierra, Andrea Hernández, Margarita Vega, Alberto Selman, Andrew F.G. Quest and Carmen Romero

Table S1. Primer sequences used for traditional and real time PCR.

Gene	Primers	Application	Amplicon	Seq
COX-2	Sense: 5′-TTC AAA GAG ATT GTG GGA AAA TTG CT-3′<br>Antisense: 5′-AGA TCA TCT CTG CCT GAG TAT CTT-3′	Traditional PCR	303 pb	NM_000963.3
Survivin	Sense: 5´-CTG GCA GCC CTT TCT CAA GGA-3´<br>Antisense: 5´-GCA ACC GGA CGA ATG CTT TT-3	Real-time PCR	225 pb	NM_001168.2
GAPDH	Sense: 5′-GAG TCA ACG GAT TTG GTC GT-3′<br>Antisense: 5′-ATC CAC AGT CTT CTG GGT G-3′	Traditional PCR	547 pb	NM_002046.6
beta-actin	Sense: 5´- TGGCAC CCA GCA CAA TGA AGA -3´<br>Antisense: 5´GAA GCA TTT GCG GTG GAC GAT 3´	Real-time PCR	166 bp	NM_001101.4

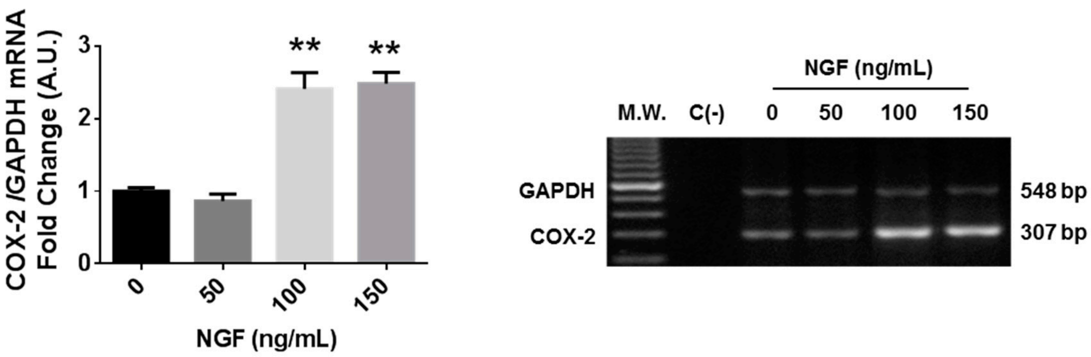

Figure S1. NGF increases COX-2 mRNA in EOC cells. A2780 cells were stimulated with NGF (50, 100 and 150 ng/mL) for 2 h and then analyzed by RT-PCR. Left: semi-quantification of COX-2 mRNA. Right: Representative image of COX-2 products in an agarose gel. n = 4. C: negative control. MW: molecular weight. ** = $p < 0.01$ with respect to basal condition. Statistical analysis: Kruskal Wallis test and Dunn´s post-test. Results are expressed as the mean ± standard error of the mean (SEM).

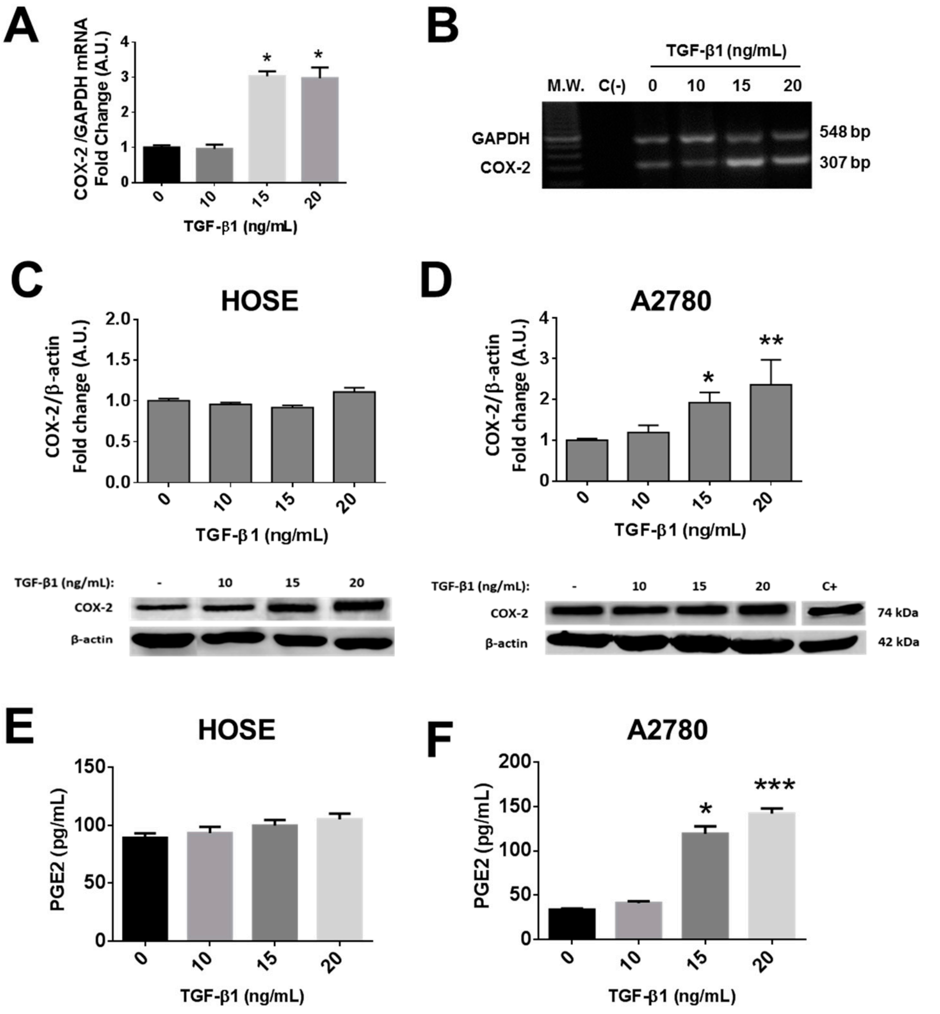

Figure S2. EOC cells respond to TGF-β stimulation increasing COX-2 and PGE_2. The ovarian cell lines HOSE and A2780 were stimulated with TGF-β (10, 15 and 20 ng/mL) for 2 h. (**A**): Semi-quantitative analysis of COX-2 mRNA levels induced by TGF-β stimulation. (**B**): A representative image of COX-2 products in an agarose gel. MW: molecular weight standard. C: negative control. (**C**–**D**): COX-2 protein levels induced by TGF-β stimulation in HOSE and A2780 cells evaluated by Western blotting. C+: extract from HEK cells that overexpress COX-2 was employed as a positive control. (**E**–**F**): PGE_2 levels in the culture supernatants of HOSE and A2780 cells, respectively, after TGF-β stimulation. n = 4. * = $p < 0.05$, ** = $p < 0.01$ and *** = $p < 0.001$ as indicated or with respect to basal condition. Statistical analysis: Kruskal Wallis test and Dunn´s post-test. Results are expressed as the mean ± standard error of the mean (SEM).

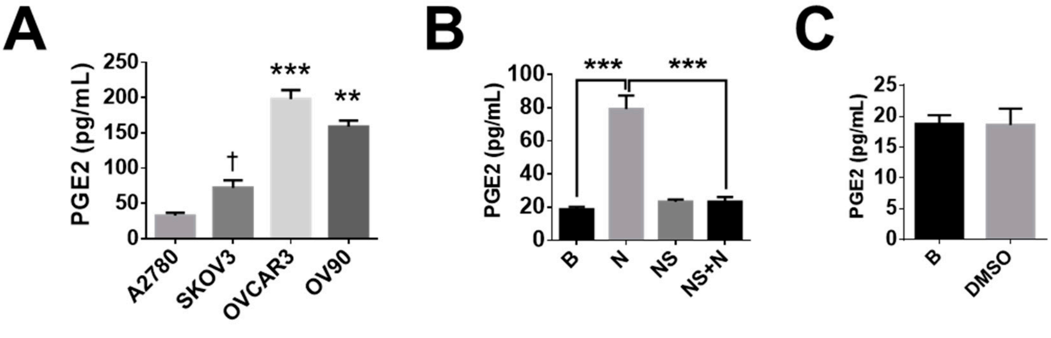

Figure S3. PGE_2 levels in media from different EOC cells before and after COX-2 inhibition. (**A**): Basal PGE_2 in the EOC cell lines A2780, SKOV3, OVCAR3 and OV90. ** = $p < 0.01$ and *** = $p < 0.001$ respect

to A2780 (Kruskal Wallis test and Dunn´s post-test). † = $p < 0.05$ respect to A2780 (Mann Whitney test). (**B**): A2780 cells were treated with the COX-2 inhibitor (NS, 20 μM) for 24 h and stimulated with NGF (N, 150 ng/mL) during the last 2 h. PGE_2 was measured in culture supernatants. (**C**): PGE_2 in culture supernatants of A2780 cells under basal conditions (B, without stimuli) or treated with DMSO (NS vehicle). $n = 4$ or more. *** = $p < 0.001$ as indicated. Statistical analysis: Kruskal Wallis test and Dunn´s post-test. Results were expressed as mean ± standard error of the mean (SEM).

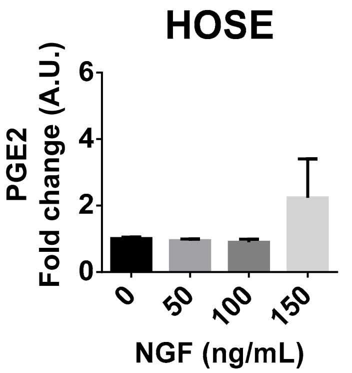

Figure S4. PGE_2 levels after NGF stimulation of HOSE cells. HOSE cells were stimulated with NGF (50, 100 and 150 ng/mL) for 2 h and PGE_2 was measured in the culture supernatants by ELISA.

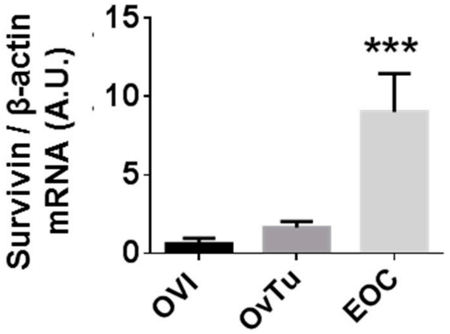

Figure S5. mRNA levels of survivin during EOC progression. Survivin mRNA was obtained from ovarian epithelium of inactive ovaries (OVI, $n = 4$), benign tumor (BeT, $n = 5$), borderline tumor (BorT, $n = 6$) and either well (EOC I, $n = 2$), moderately (EOC II, $n = 5$) or poorly differentiated (EOC III, $n = 8$) ovarian cancers. Then, samples were analyzed by real time PCR. *** = $p < 0.01$ vs. OVI. Statistical analysis: Kruskal Wallis test and Dunn´s post-test. Results are expressed as the mean ± standard error of the mean (SEM).

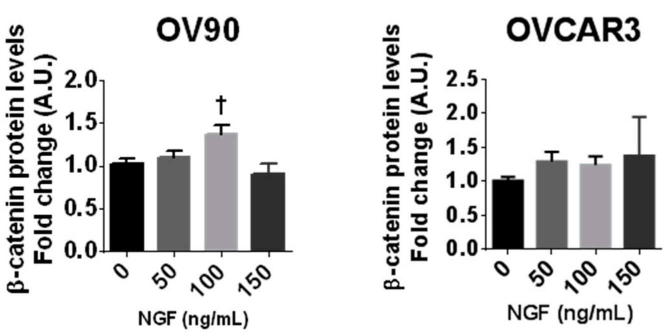

Figure S6. Effect of NGF on β-catenin protein levels in EOC cells. OV90 and OVCAR3 cells were serum-deprived for 24 h and then stimulated with NGF (0, 50, 100 and 150 ng/mL) for 8 h. $n = 4$ or more. † = $p < 0.05$ with respect to the basal condition (Mann Whitney test).

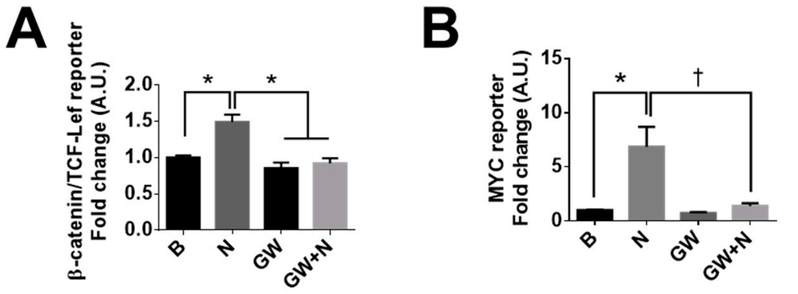

Figure S7. β-catenin/TCF-Lef and MYC transcriptional activity in A2780 cells. β-catenin/TCF-Lef (**A**) and MYC transcriptional activity (**B**) were measured in gene-reporter assays as described. B = basal condition (without stimuli), N = NGF (100 ng/mL), GW: GW441756 (specific TRKA inhibitor). $n = 4$ or more. * = $p < 0.05$ (Kruskal Wallis test and Dunn´s post-test) and † = $p < 0.05$ (Mann Whitney test) as indicated. Results are expressed as the mean ± standard error of the mean (SEM).

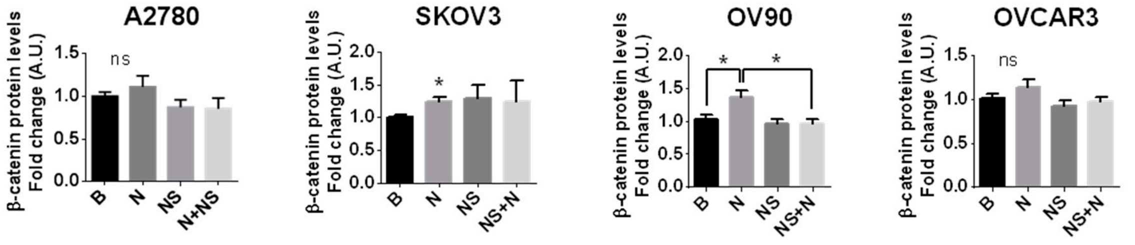

Figure S8. β-catenin protein levels after NGF stimulation and NS398 treatment of EOC cells. EOC cells were treated with the specific COX-2 inhibitor (NS, 20 μM, 24 h) and/or NGF (N; 150 ng/mL for 2h in SKOV3 cells; 100 ng/mL for 8 h in OV90 cells and 150 ng/mL for 8 h in OVCAR3 cells) and β-catenin levels were then assessed by western blotting and scanning densitometry. $n = 4$ or more. * = p < 0.05 with respect to basal condition (B, without stimuli). Statistical analysis: Kruskal Wallis test and Dunn´s post-test. Results are expressed as the mean ± standard error of the mean (SEM).

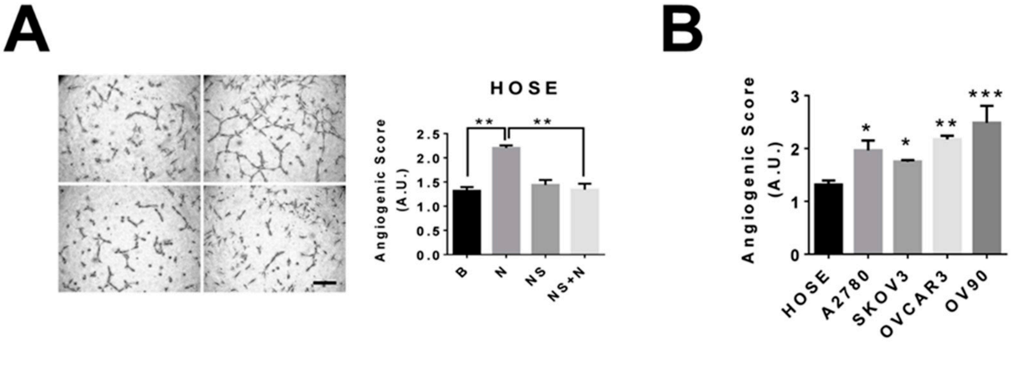

Figure S9. Angiogenic score of HOSE cells after NGF stimulation and NS treatment. (**A**): HOSE cells were stimulated with NGF (N, 100 ng/mL, 2h) and/or treated with the specific COX-2 inhibitor (NS, 20 μM, 24h). Then, culture supernatants were employed in tubule formation assays in matrigel using endothelial cells. Bar = 100 μm (**B**): basal angiogenic score of the different cell lines used. * = $p < 0.05$ and $p < 0.01$ with respect to the basal condition (without stimuli) or as indicated. Statistical analysis: Kruskal Wallis test and Dunn´s post-test. Results are expressed as the mean ± standard error of the mean (SEM).

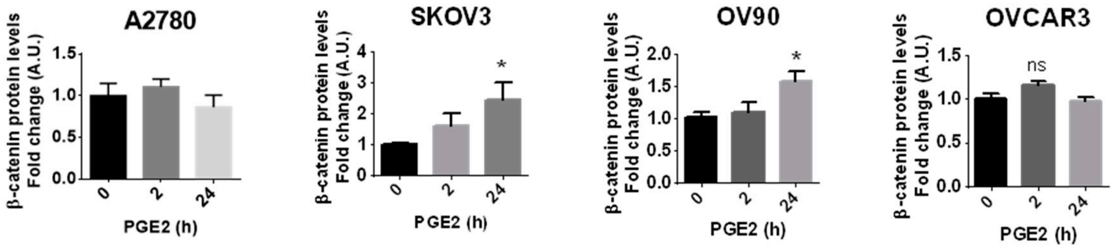

Figure S10. β-catenin protein levels after PGE_2 stimulation of EOC cells. EOC cells were serum-deprived for 24 h and then stimulated with PGE_2 (20 μM, 2 and 24 h). β-catenin levels were assessed by western-blotting and scanning densitometry. $n = 4$ or more. * = $p < 0.05$ with respect to the basal condition (without stimuli). ns: statistically not significant. Statistical analysis: Kruskal Wallis test and Dunn´s post-test. Results are expressed as the mean ± standard error of the mean (SEM).

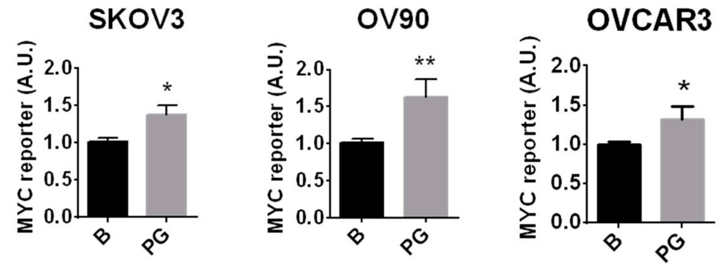

Figure S11. PGE_2 increases MYC transcriptional activity in EOC cells. EOC cells were incubated with PGE_2 (PG, 20 μM) for 24 h and reporter activity was measured. $n = 4$, * = $p < 0.05$ and $p < 0.01$ (Mann Whitney test). Results are expressed as the mean ± standard error of the mean (SEM).

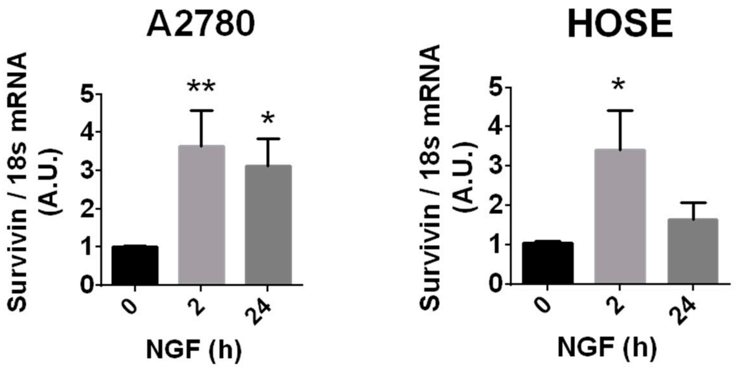

Figure S12. Survivin mRNA post- NGF stimulation in ovarian cells. A2780 and HOSE cells were stimulated with NGF (100 ng/mL, 2 or 24 h) and survivin mRNA was detected by qPCR. $n = 4$ independent experiments in duplicate. ** = $p < 0.01$ and *** = $p < 0.001$. Statistical analysis: Kruskal Wallis test and Dunn´s post-test. Results are expressed as the mean ± standard error of the mean (SEM).

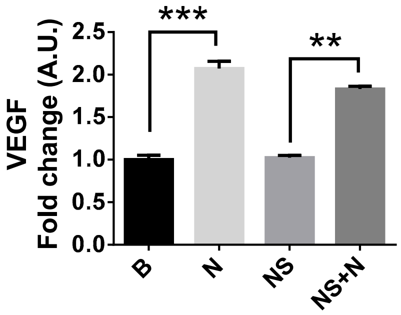

Figure S13. Effect of COX-2 inhibition for short periods of time on VEGF liberation by EOC cells. A2780 cells were incubated with the COX-2 specific inhibitor (NS, 20 μM) and stimulated with NGF (N, 150 ng/mL) for 2 h. Then, VEGF protein levels in the culture supernatants were measured. $n = 4$. ** = $p < 0.01$ and *** = $p < 0.001$. Statistical analysis: Kruskal Wallis test and Dunn´s post-test. Results are expressed as the mean ± standard error of the mean (SEM).

© 2019 by the authors. Licensee MDPI, Basel, Switzerland. This article is an open access article distributed under the terms and conditions of the Creative Commons Attribution (CC BY) license (http://creativecommons.org/licenses/by/4.0/).
