# Supplementary material for: NGF-Enhanced Vasculogenic Properties of Epithelial Ovarian Cancer Cells Is Reduced by Inhibition of the COX-2/PGE2 Signaling Axis
Source: Cancers (Basel). 2019 Dec 7;11(12):1970. doi: 10.3390/cancers11121970 (PMC6966471; doi:10.3390/cancers11121970)
Supplement: Supplementary file 1 [file cancers-11-01970-s001.zip › Figure S14. Panel of original captures.pdf]

Supplementary material

Set of original captures of western-blots

The membrane had to be cut into several segments to visualize the different proteins evaluated. Stripping was not an option due to the difficulty in visualizing some of these proteins. We present a set of original western-blot captures which were used to obtain the graphs of the manuscript.

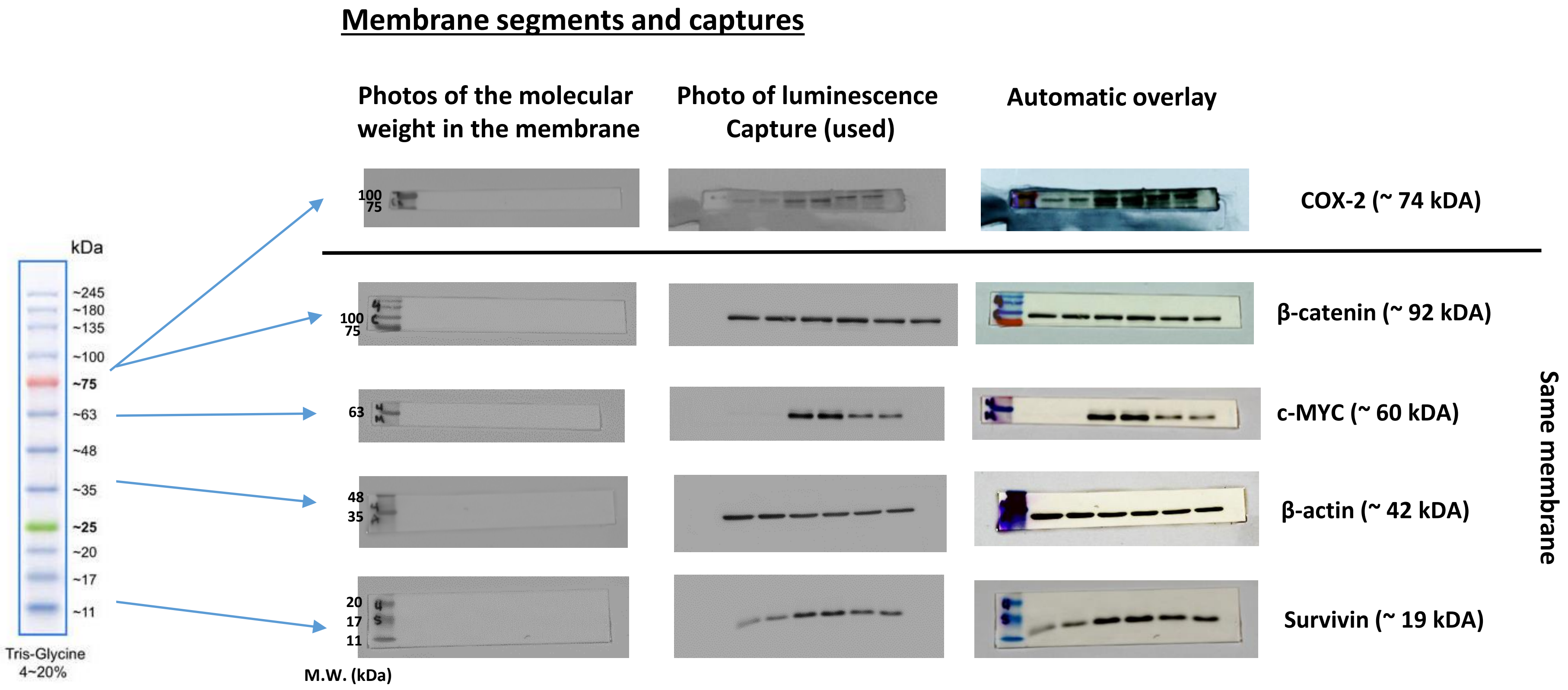

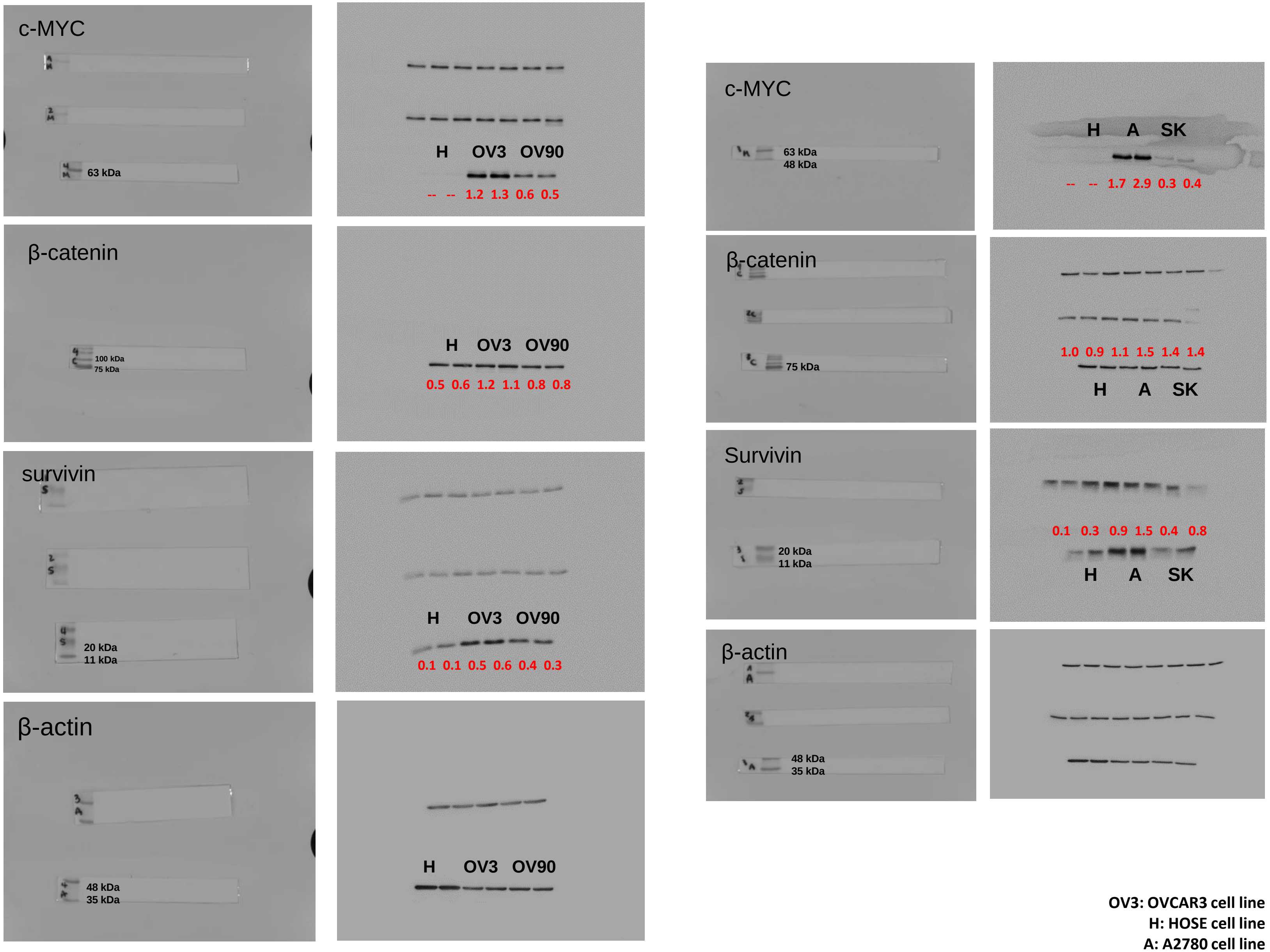

OV3: OVCAR3 cell line
H: HOSE cell line
A: A2780 cell line
SK: SKOV3 cell line

In red: protein/β-actin ratio

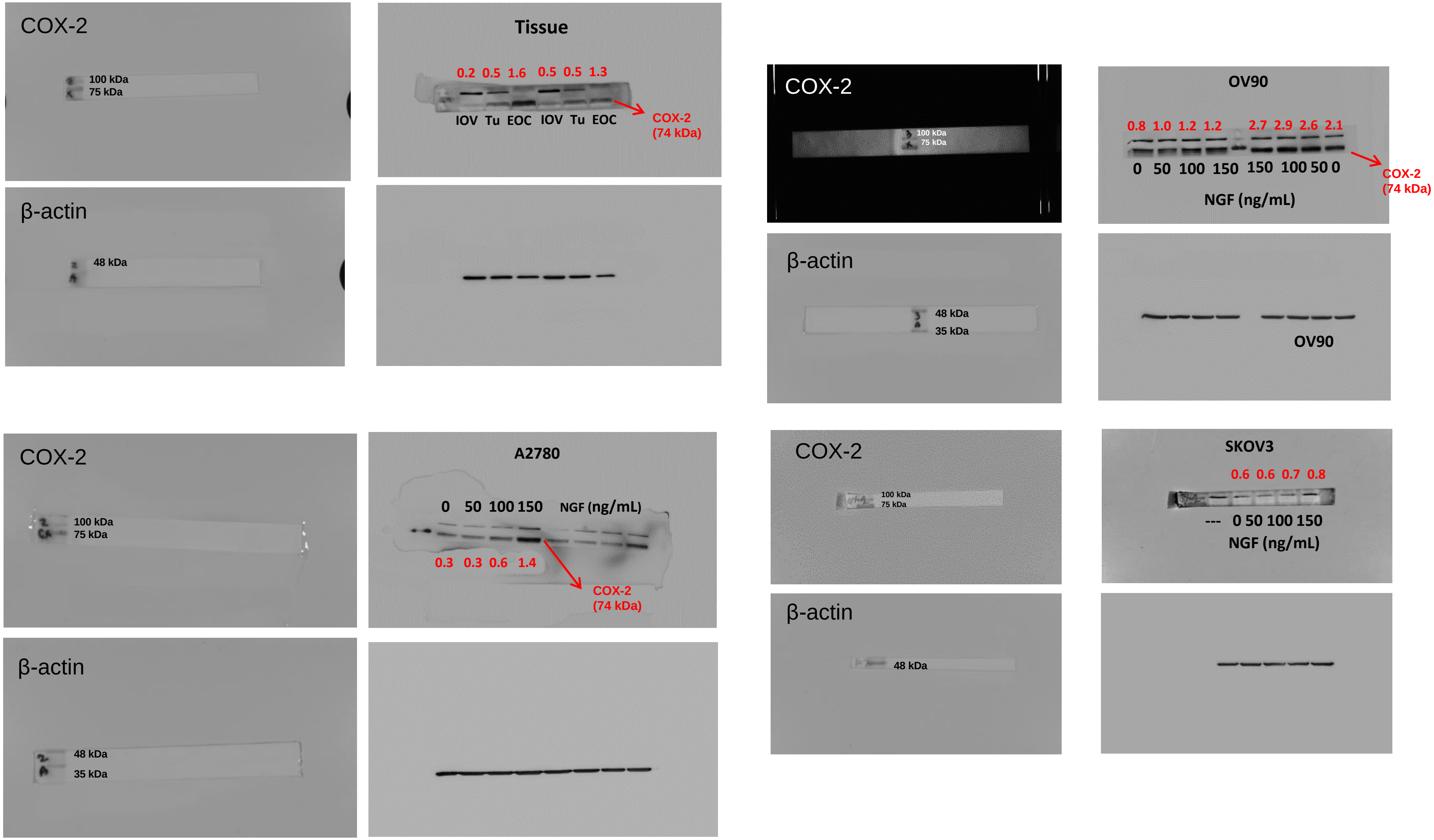

OV3: OVCAR3 cell line
IOV: inactive ovary (non-tumoral)
Tu: ovarian tumor
EOC: epitelial ovarian cancer

In red: protein/β-actin ratio

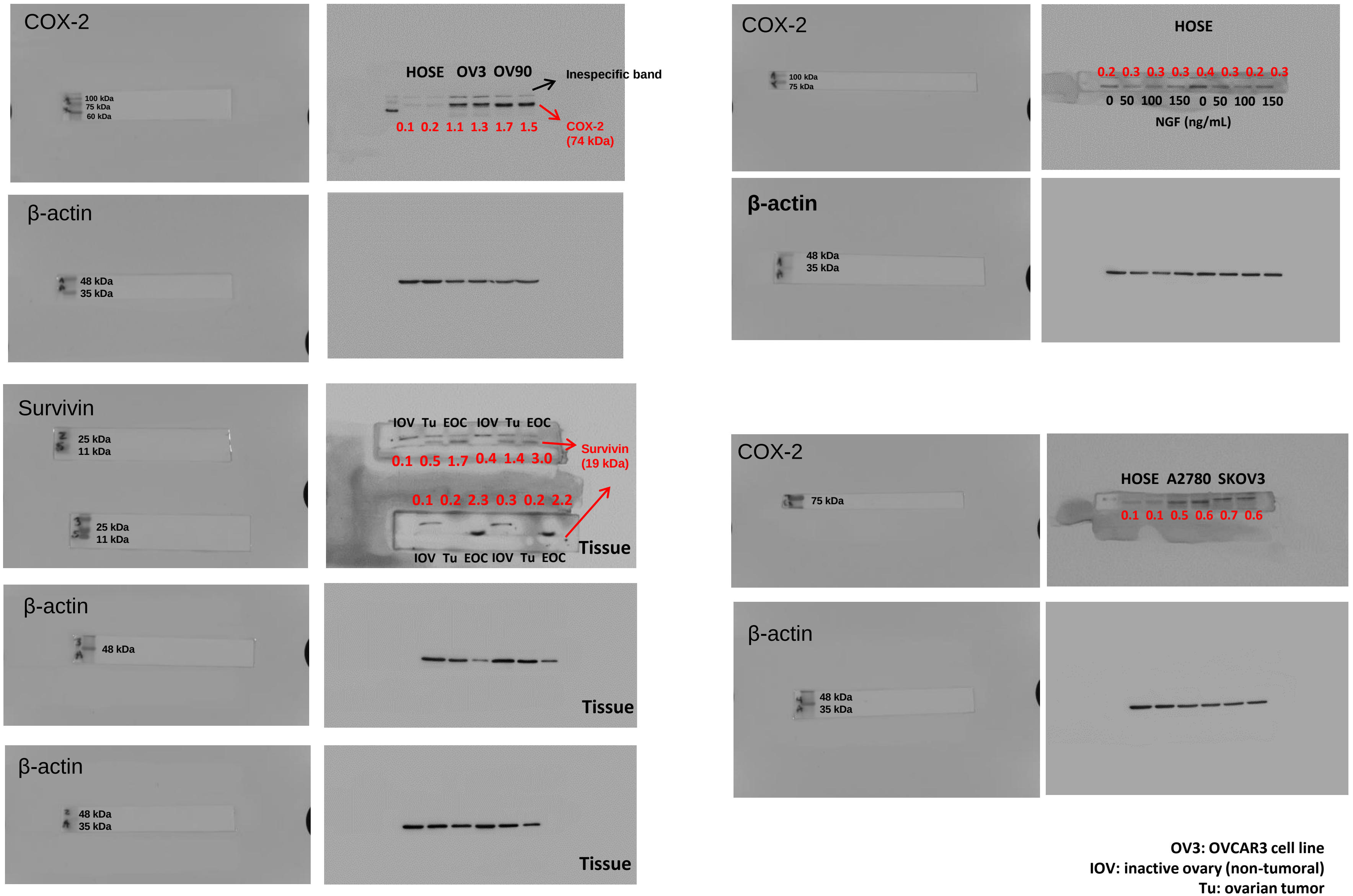

OV3: OVCAR3 cell line
IOV: inactive ovary (non-tumoral)
Tu: ovarian tumor
EOC: epithelial ovarian cancer

In red: protein/β-actin ratio

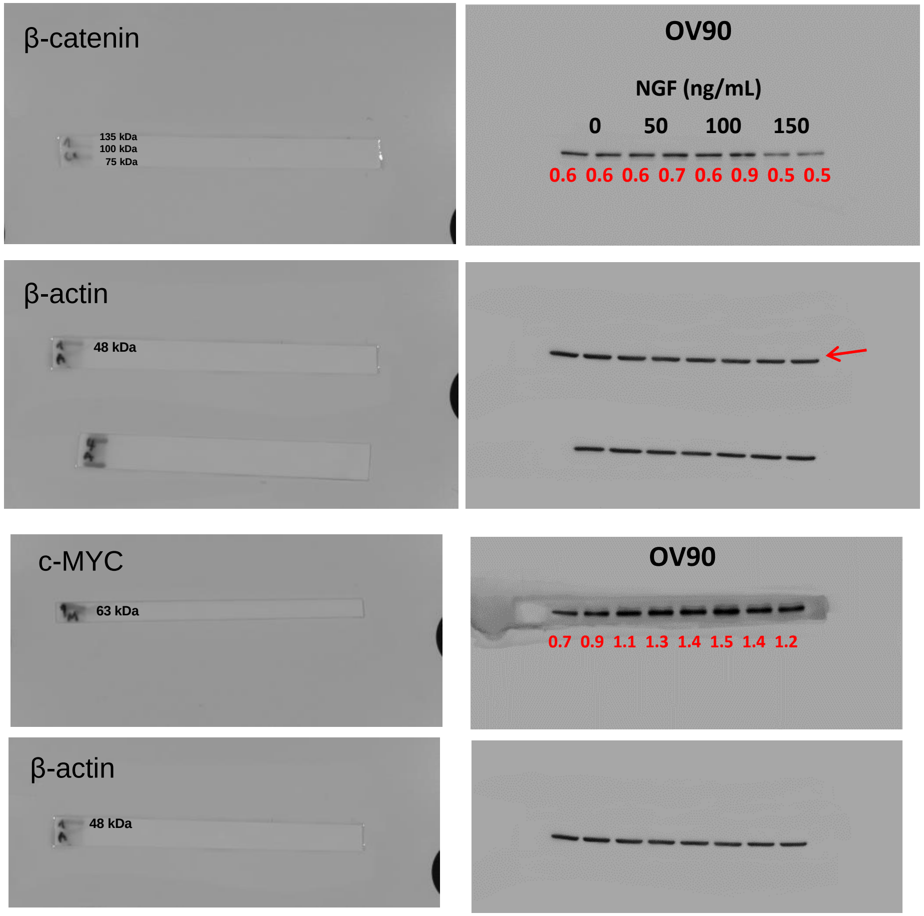

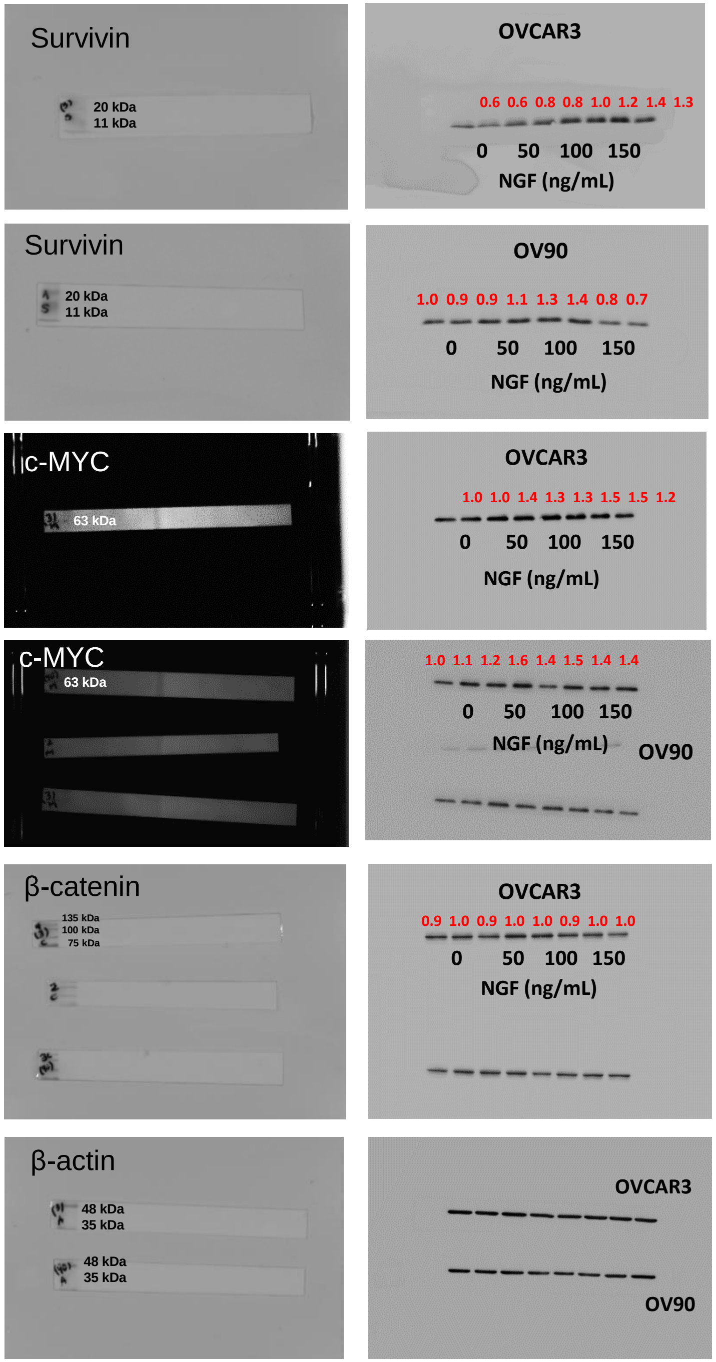

OV3: OVCAR3 cell line
IOV: inactive ovary (non-tumoral)
Tu: ovarian tumor
EOC: epithelial ovarian cancer

In red: protein/β-actin ratio

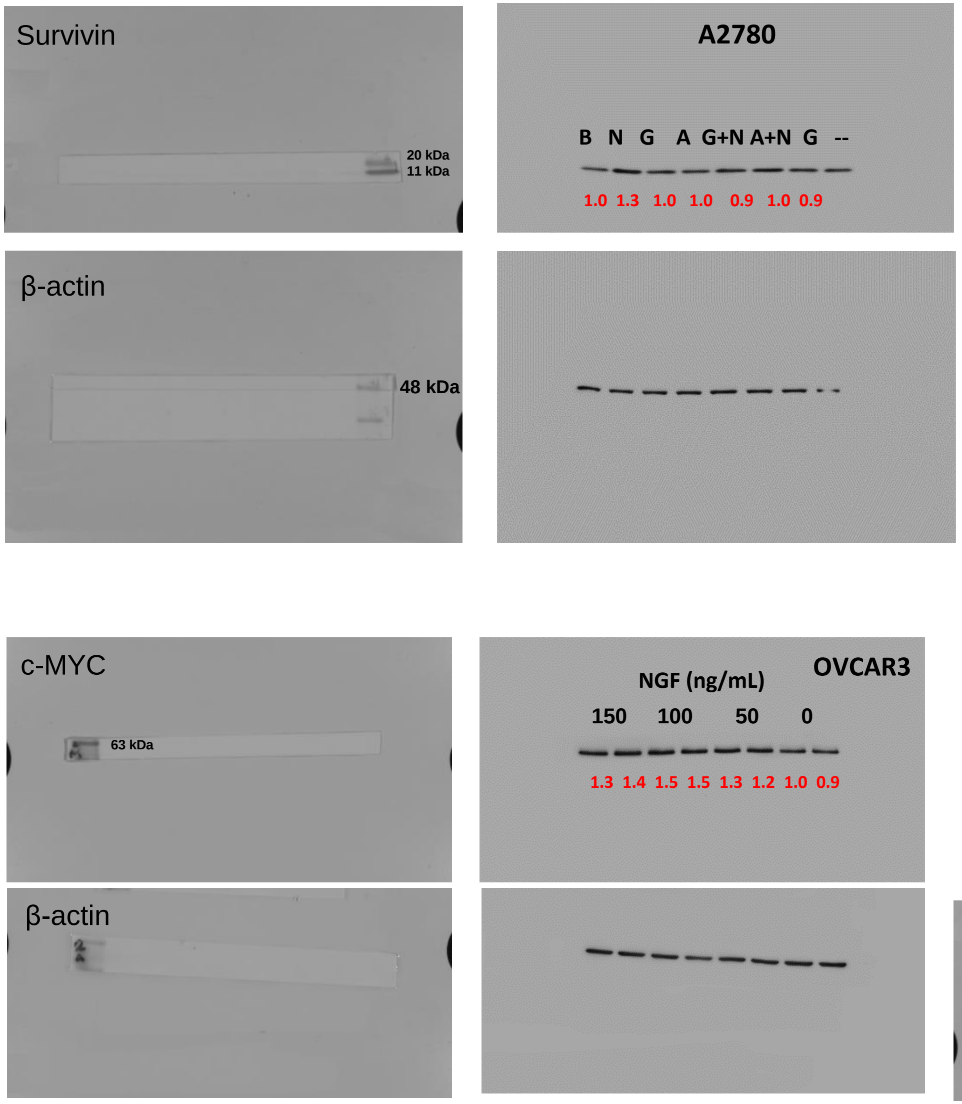

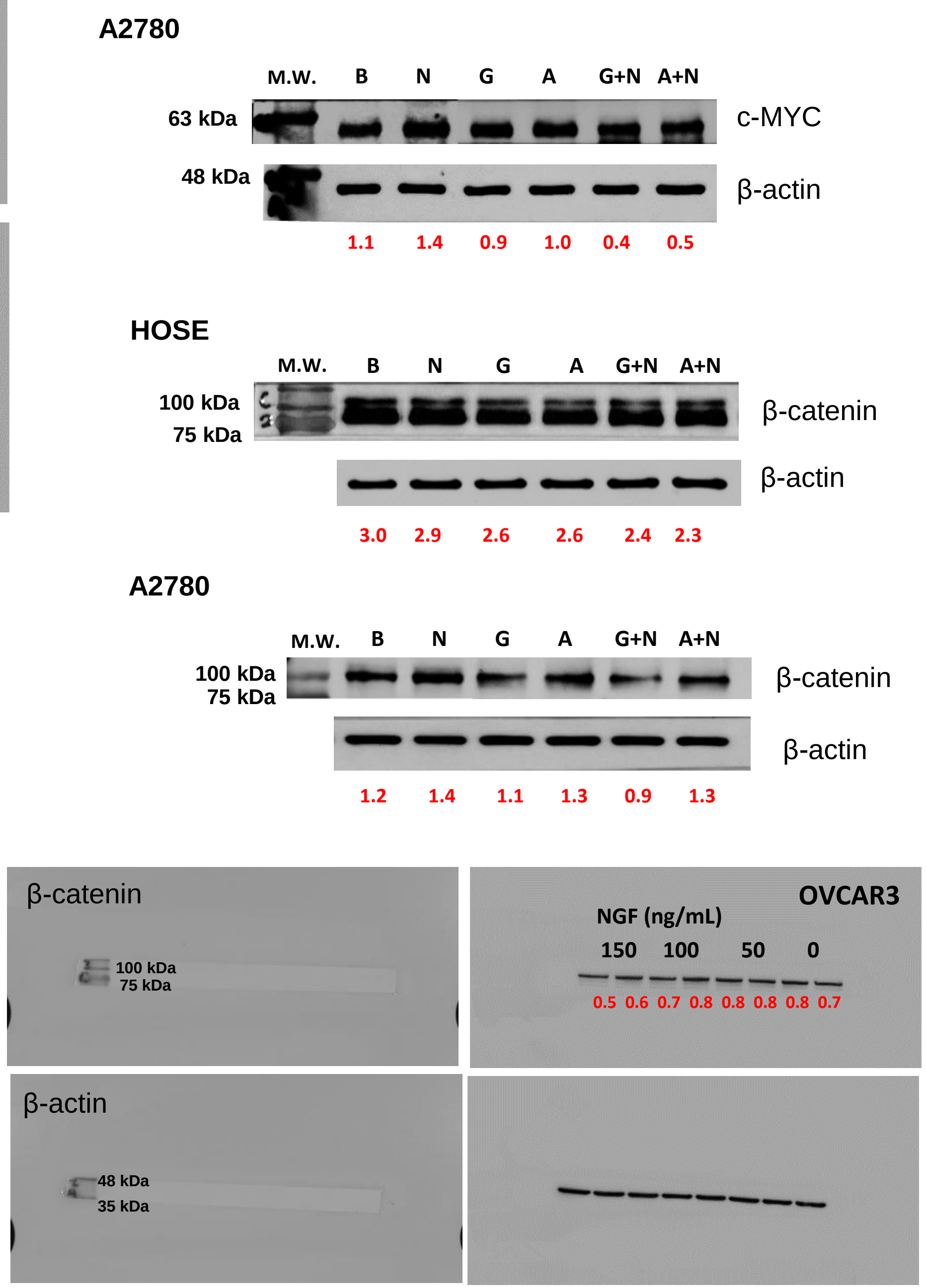

M.W.: molecular weight
B: basal
N: NGF
G: GW441756 (trkA inhibitor)
A: anti-NGF antibody

In red: protein/β-actin ratio

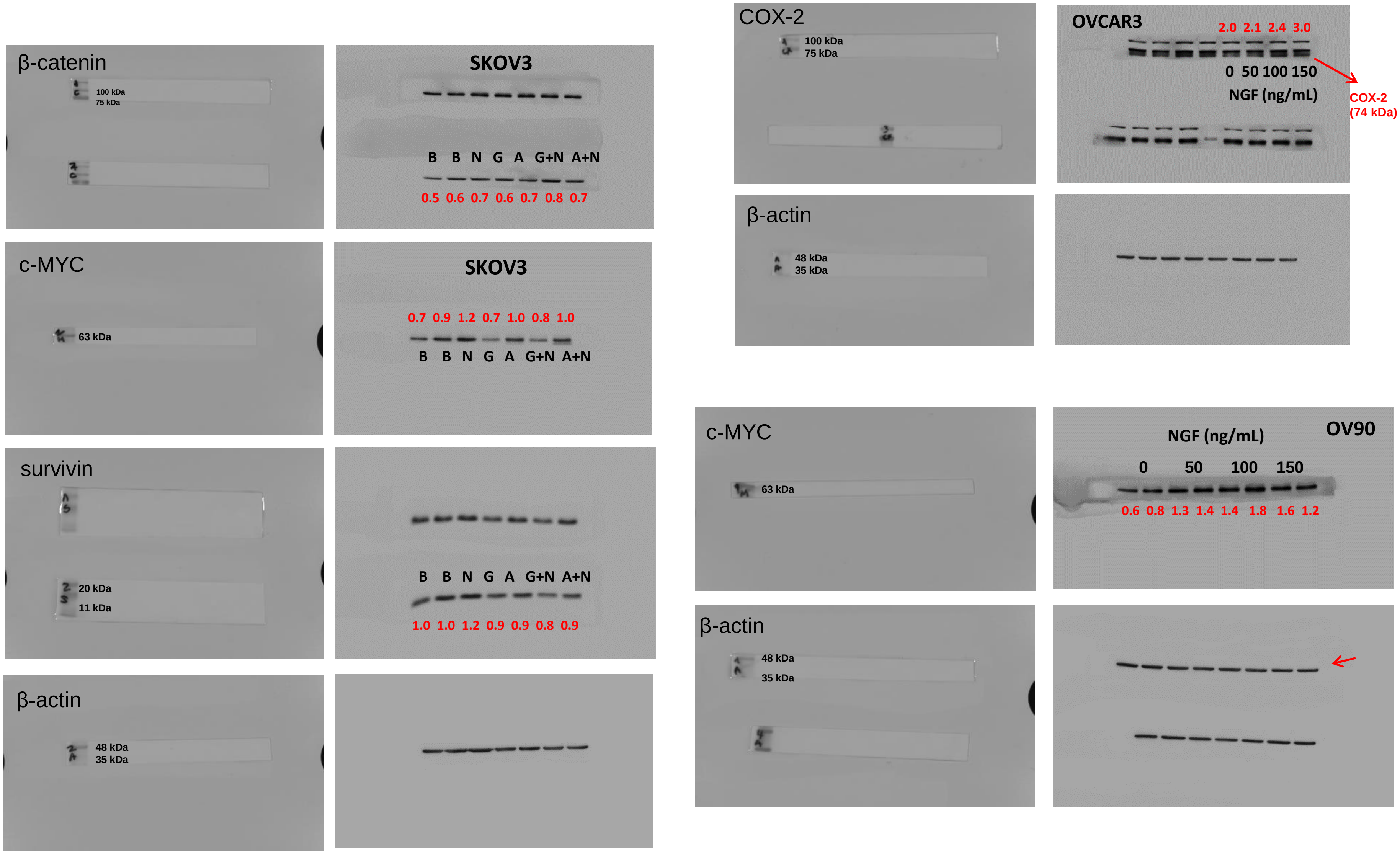

B: basal
N: NGF
G: GW441756 (trkA inhibitor)
A: anti-NGF antibody

In red: protein/β-actin ratio

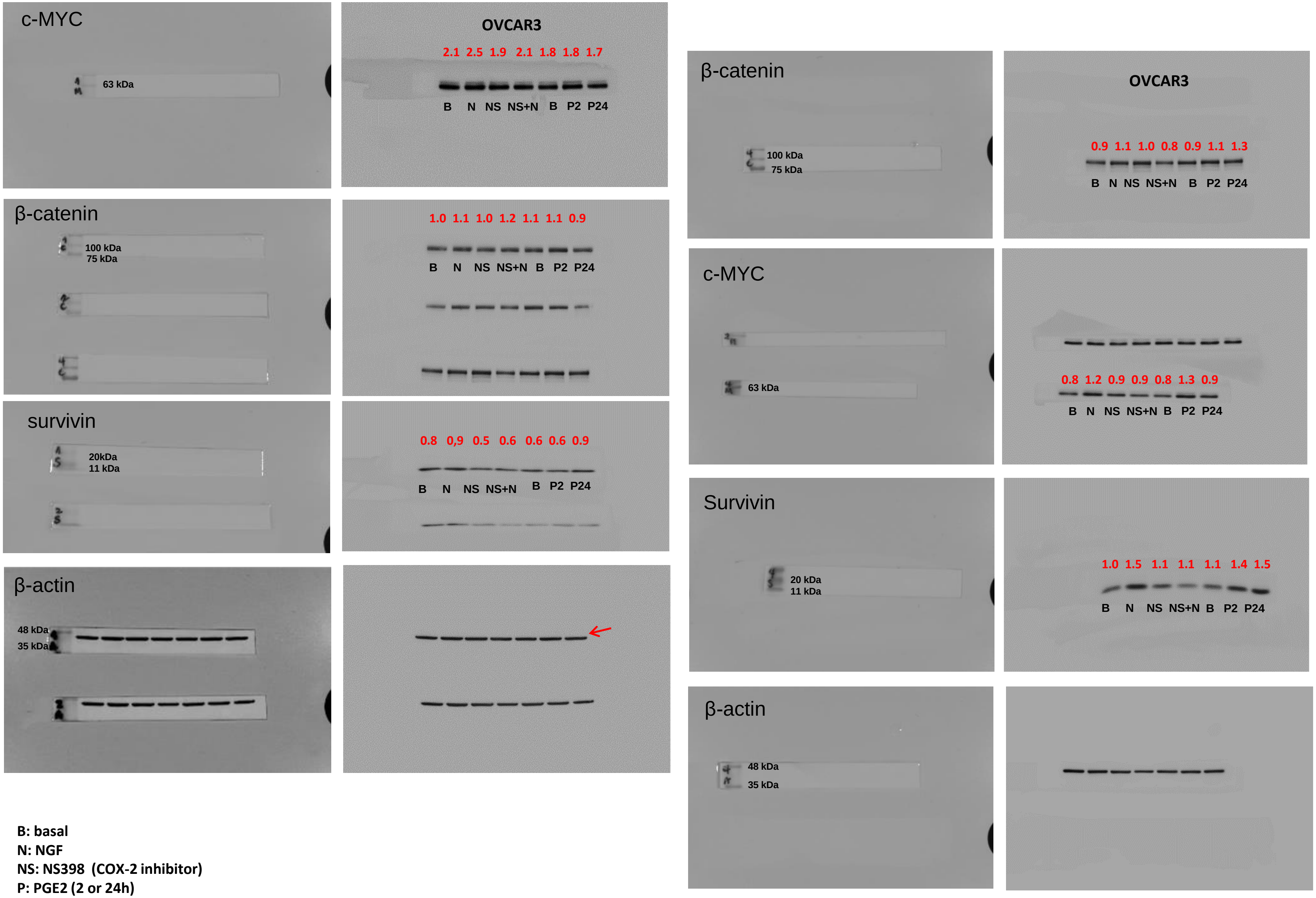

B: basal
N: NGF
NS: NS398 (COX-2 inhibitor)
P: PGE2 (2 or 24h)

In red: protein/β-actin ratio

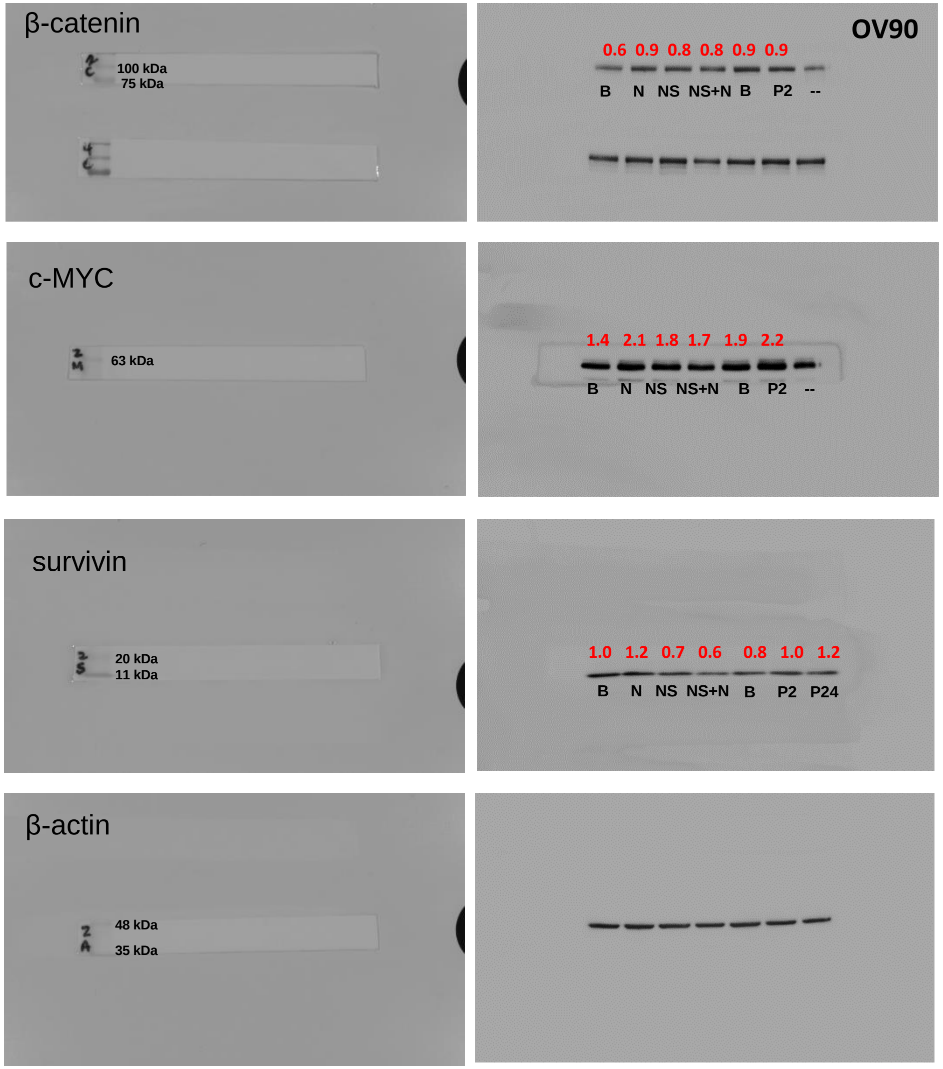

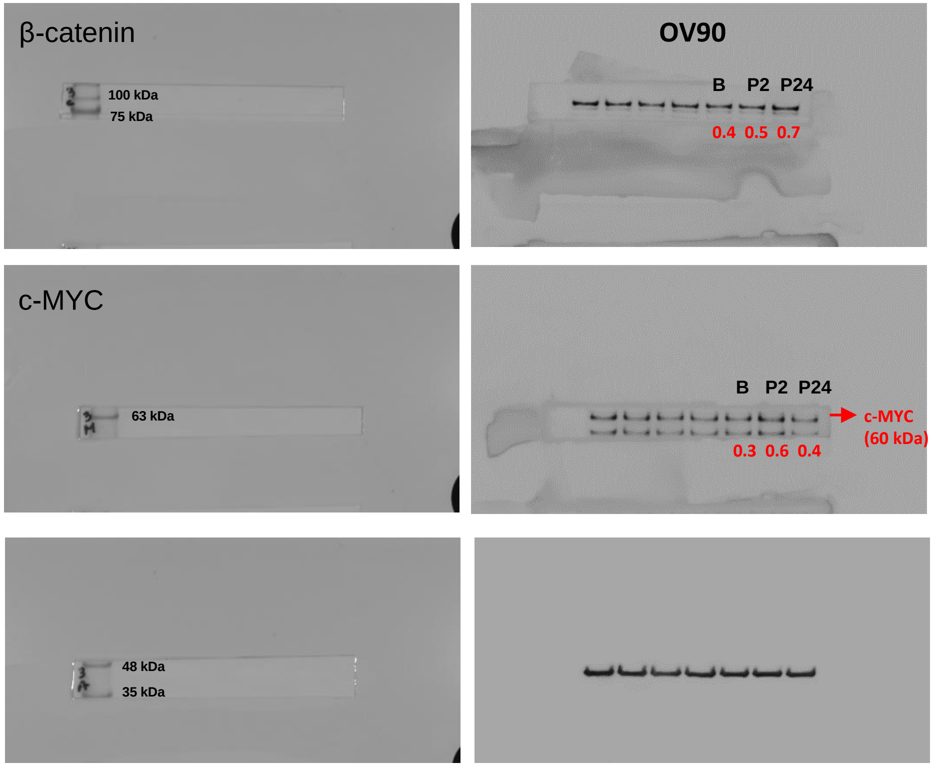

B: basal
N: NGF
NS: NS398 (COX-2 inhibitor)
P: PGE2 (2 or 24h)

In red: protein/β-actin ratio

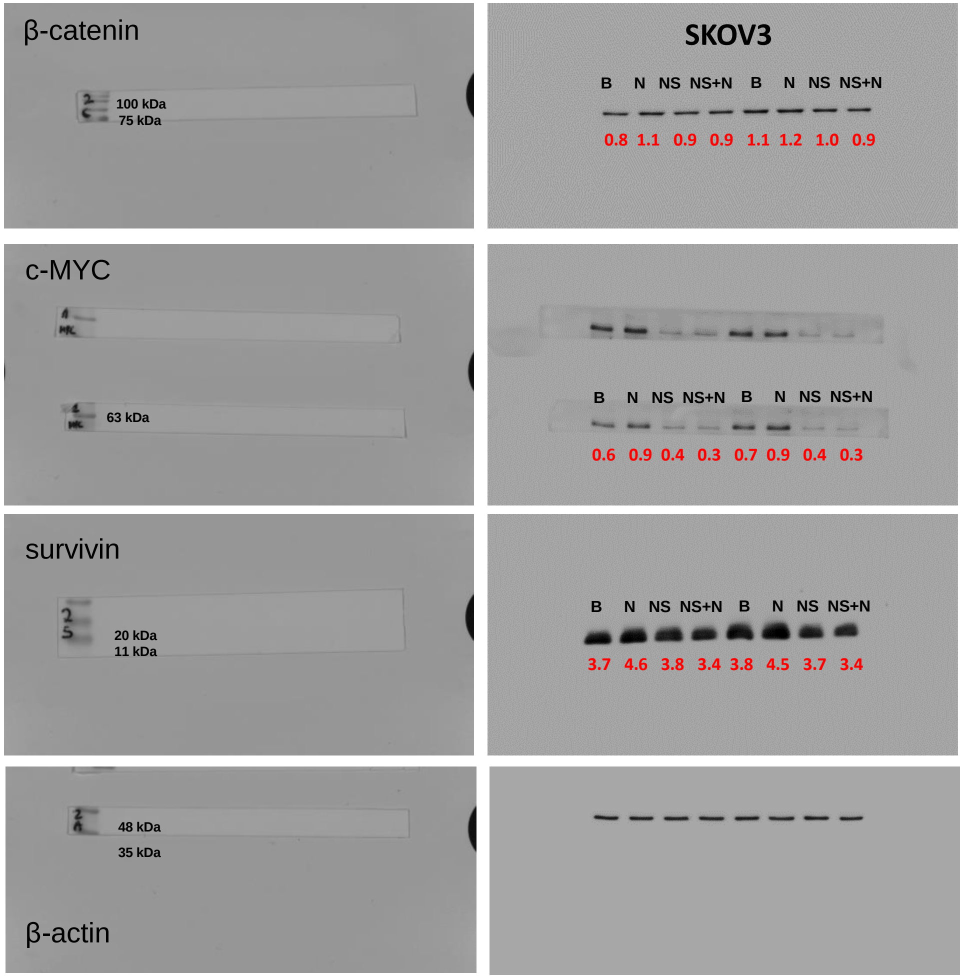

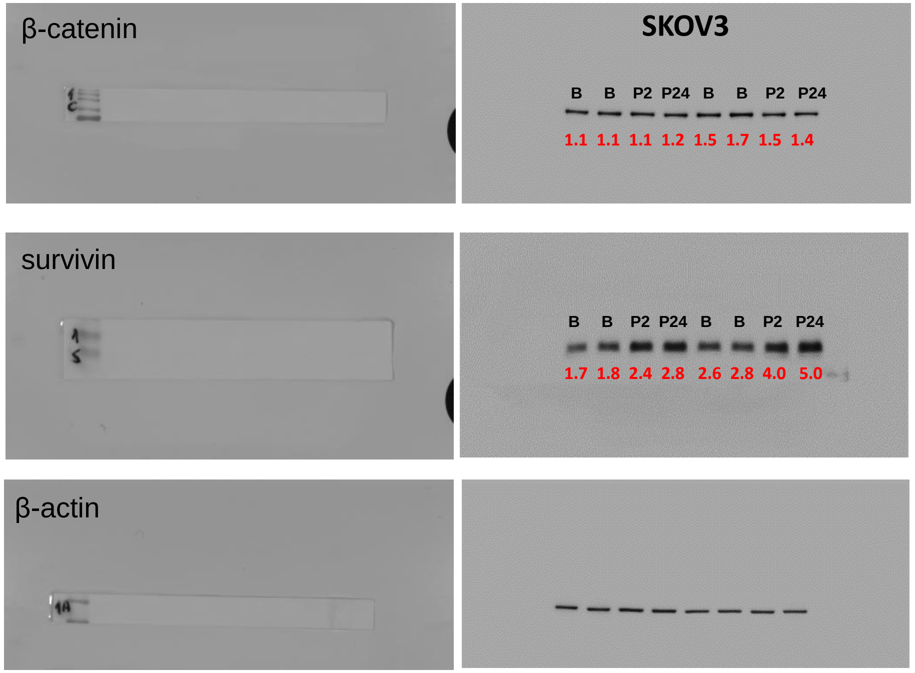

B: basal
N: NGF
NS: NS398 (COX-2 inhibitor)
P: PGE2 (2 or 24h)

In red: protein/β-actin ratio

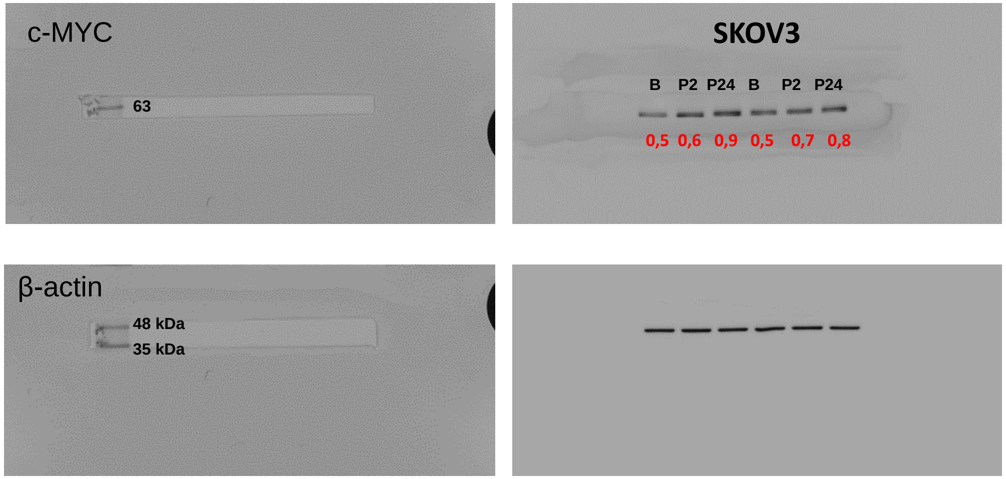

A2780

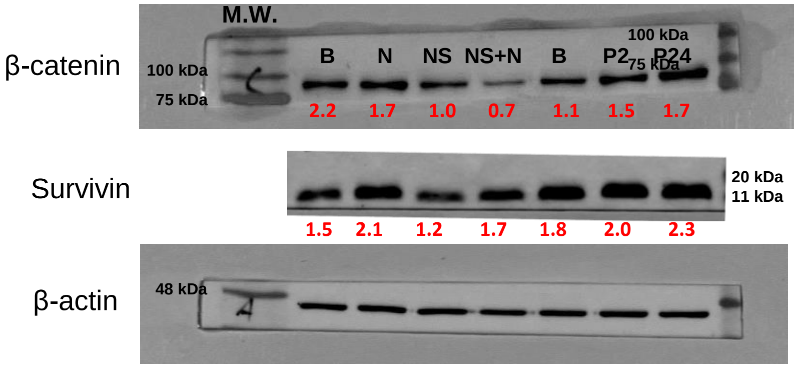

A2780

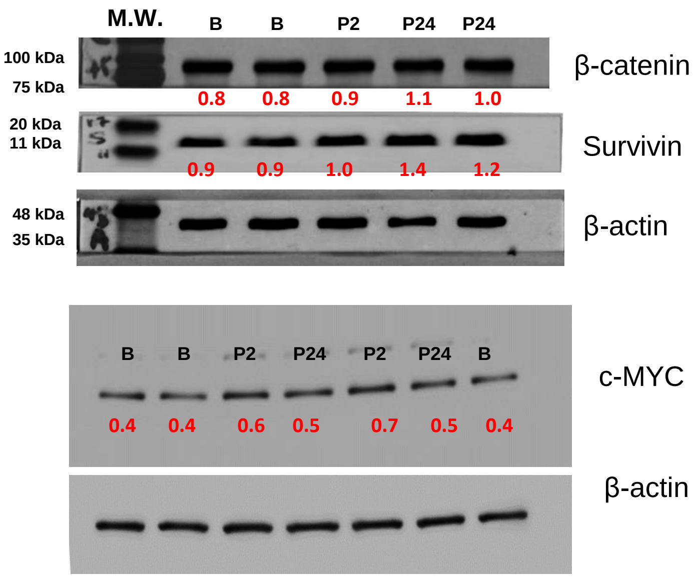

B: basal
N: NGF
NS: NS398 (COX-2 inhibitor)
P: PGE2 (2 or 24h)

In red: protein/β-actin ratio
